# Hospital Discharge Interventions: A Cost-Savings Comparison

**DOI:** 10.1093/geroni/igaa057.450

**Published:** 2020-12-16

**Authors:** Bonnie Albright, Yael Pelenur

**Affiliations:** 1 University of Massachusetts - Boston, Boston, Massachusetts, United States; 2 University of Vermont, Burlington, Vermont, United States

## Abstract

For an older patient, transitioning back into the community after an acute health incident is a critical juncture. Avoidable acute-care readmissions are expensive for hospitals and federal programs. Negative health outcomes for patients and negative financial outcomes for hospitals and federal programs have focused attention on effective discharge interventions to improve care transitions and decrease avoidable acute-care readmissions. This study compares cost and readmissions outcomes from peer-reviewed publication data for three discharge interventions: Care Transitions Intervention (CTI), Project RED (ReEngineered Discharge), and the Transitional Care Model (TCM). This study adjusted costs to 2015 rates and compared cost savings per patient, return on investment (ROI) and percent reduction of readmissions. Cost savings per patient (2015-adjusted) were found for all interventions: CTI ($152.89); Project RED ($327.03); TCM ($1565.84). ROI was positive for all interventions: CTI (832%); Project RED (535%); TCM (232%). Compared to control groups, intervention group readmissions were 3.6% lower for CTI (n.s.), 5.5% lower for Project RED (p<.05), and 13.2% lower for TCM (p<.05). These three discharge interventions differ in scale and intensity, but they all show cost savings and reductions in readmissions. The lower-cost intervention shows cost savings and ROI (CTI), and the more resource-intensive interventions (Project RED and TCM) reduce costs and statistically significantly reduce rates of readmission. Even with small budget dollars, hospitals have options for finding an effective discharge intervention to reduce costs and readmission rates.

